# Supplementary material for: Lead yourself to the zone and be happy: The effect of self-leadership development on flow and happiness
Source: PLoS One. 2025 Sep 9;20(9):e0331673. doi: 10.1371/journal.pone.0331673 (PMC12419596; doi:10.1371/journal.pone.0331673)
Supplement: S1 Table — (DOCX) [file pone.0331673.s001.docx]

**Supporting Table 1** Parameter estimates and 95% confidence intervals (CI) for the effect of between- and within-subject self-leadership on situational flow and happiness data collected using ESM, while controlling for time slot of data collection, sociodemographic and work-related variables

|  | **Situational flow** | | |  | | **Happiness** | | | |  |
| --- | --- | --- | --- | --- | --- | --- | --- | --- | --- | --- |
| **Fixed effects** | Estimates | 95% CI | *p* | |  | | Estimates | 95% CI | *p* | |
| (Intercept) | 3.83 | 3.58 – 4.09 | **< .001** | |  | | 3.54 | 3.29 – 3.78 | **< .001** | |
| Day | 0.00 | 0.00 – 0.01 | **< .001** | |  | | 0.00 | -0.00 – 0.00 | **.150** | |
| Time slot: 1-5 p.m. | 0.05 | 0.00 – 0.09 | **.050** | |  | | 0.04 | -0.00 – 0.08 | **.054** | |
| Time slot: 5-8 p.m. | 0.02 | -0.03 – 0.06 | .508 | |  | | 0.05 | 0.01 – 0.09 | **.014** | |
| Between-subjects self-leadership | 0.43 | 0.18 – 0.69 | **< .001** | |  | | 0.46 | 0.21 – 0.70 | **< .001** | |
| Within-subject self-leadership | 0.11 | -0.07 – 0.28 | .233 | |  | | 0.14 | -0.06 – 0.34 | .158 | |
| Sex: female | 0.16 | -0.06 – 0.38 | .152 | |  | | 0.01 | -0.20 – 0.22 | .936 | |
| Age group: > 40y | 0.13 | -0.07 – 0.34 | .195 | |  | | -0.09 | -0.29 – 0.10 | .349 | |
| Educational level: postgraduate studies | -0.06 | -0.25 – 0.12 | .489 | |  | | -0.20 | -0.38 – -0.02 | **.028** | |
| Seniority in the company: ≥ 10y | 0.05 | -0.15 – 0.24 | .648 | |  | | 0.09 | -0.10 – 0.28 | .343 | |
| Seniority in the function: > 2y | 0.14 | -0.06 – 0.34 | .176 | |  | | 0.04 | -0.16 – 0.23 | .707 | |
| Weekly overtime: > 5h | 0.11 | -0.07 – 0.30 | .222 | |  | | 0.09 | -0.09 – 0.26 | .335 | |
| Working schedule flexibility: high | -0.08 | -0.27 – 0.11 | .403 | |  | | -0.07 | -0.25 – 0.11 | .417 | |
| Observations | 4177 |  |  | |  | | 4177 |  |  | |
| Marginal *R^2^* / Conditional *R^2^* | 0.08 / 0.31 |  |  | |  | | 0.08 / 0.43 |  |  | |
