# Supplementary material for: Lead yourself to the zone and be happy: The effect of self-leadership development on flow and happiness
Source: PLoS One. 2025 Sep 9;20(9):e0331673. doi: 10.1371/journal.pone.0331673 (PMC12419596; doi:10.1371/journal.pone.0331673)
Supplement: S2 Table — (DOCX) [file pone.0331673.s002.docx]

**Supporting Table 2** Variation of self-leadership, situational and dispositional flow, and happiness over the study period provided as estimates for each time of assessment.

|  | **Estimate** | **SE** | **Lower CL** | **Upper CL** |
| --- | --- | --- | --- | --- |
| **Self-leadership** |  |  |  |  |
| Time 1 | 4.05 | 0.05 | 3.95 | 4.16 |
| Time 2 | 4.16 | 0.05 | 4.06 | 4.26 |
| Time 3 | 4.30 | 0.05 | 4.20 | 4.40 |
| Time 4 | 4.34 | 0.05 | 4.24 | 4.44 |
| **Situational flow** |  |  |  |  |
| Time 1 | 4.04 | 0.06 | 3.92 | 4.15 |
| Time 2 | 4.05 | 0.06 | 3.94 | 4.17 |
| Time 3 | 4.16 | 0.06 | 4.04 | 4.27 |
| Time 4 | 4.18 | 0.06 | 4.07 | 4.30 |
| **Dispositional flow** |  |  |  |  |
| Time 1 | 4.24 | 0.06 | 4.12 | 4.35 |
| Time 2 | 4.27 | 0.06 | 4.15 | 4.38 |
| Time 3 | 4.30 | 0.06 | 4.19 | 4.41 |
| Time 4 | 4.28 | 0.06 | 4.17 | 4.40 |
| **Happiness** |  |  |  |  |
| Time 1 | 3.49 | 0.06 | 3.38 | 3.60 |
| Time 2 | 3.44 | 0.06 | 3.33 | 3.56 |
| Time 3 | 3.56 | 0.06 | 3.45 | 3.67 |
| Time 4 | 3.64 | 0.06 | 3.52 | 3.75 |

SE, Standard Error; CL, Confidence Limit
