# Supplementary figures and images for: Lead yourself to the zone and be happy: The effect of self-leadership development on flow and happiness
Source: PLoS One. 2025 Sep 9;20(9):e0331673. doi: 10.1371/journal.pone.0331673 (PMC12419596; doi:10.1371/journal.pone.0331673)

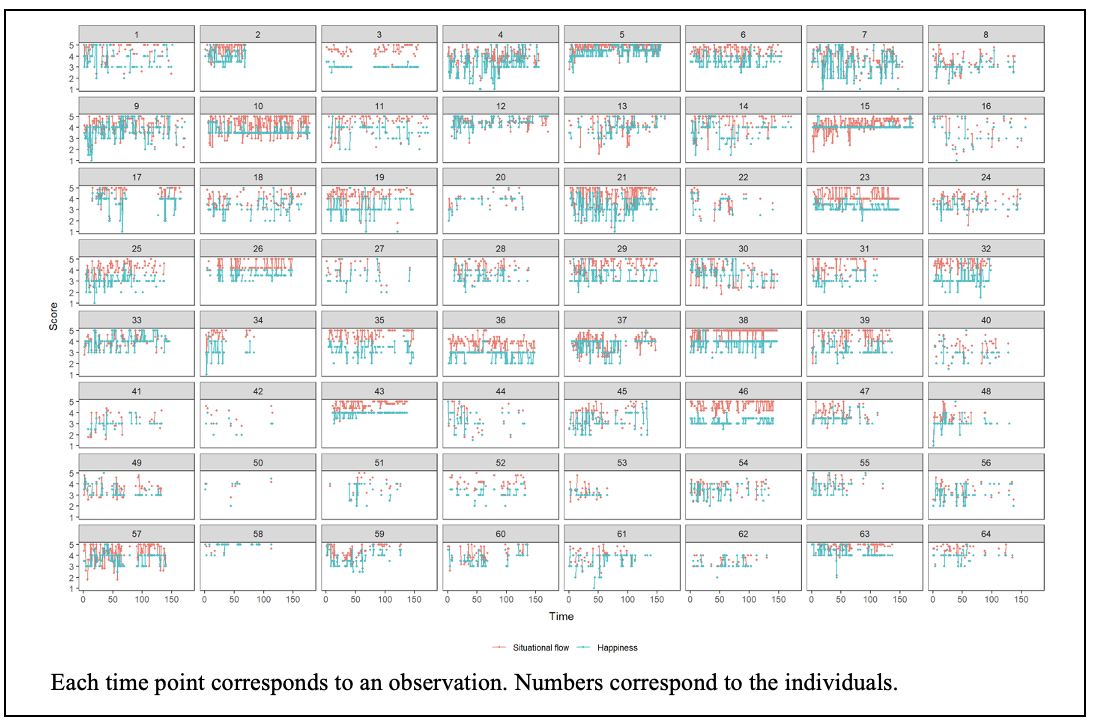

Supplement: S1 Fig — (TIFF) [file pone.0331673.s003.tiff]
