# Supplementary material for: Lead yourself to the zone and be happy: The effect of self-leadership development on flow and happiness
Source: PLoS One. 2025 Sep 9;20(9):e0331673. doi: 10.1371/journal.pone.0331673 (PMC12419596; doi:10.1371/journal.pone.0331673)
Supplement: Attachment — Submitted filename: Plos One.docx [file pone.0331673.s004.docx]

Thanks for providing the opportunity to review this paper.

The manuscript examines the relationship between self-leadership, flow and happiness at the workplace. The study also examines the mediating effect of flow on the relationship between self-leadership and happiness.

The study is opportune and relevant in the context of the current unpredictable times, at the individual and the organizational level. The paper is well written and presents fluid thinking. However, I have a few suggestions that the authors may consider:

1. Introduction

I tried looking for the research gap that the authors are trying the bridge. It was difficult to identify the research context in the lines 122-126 that the work is attempting to fulfil. To my mind, this should be brought forward in the structure of the study.

At times, the overall argumentation was in itself answering the question that the authors are trying to respond. For example, in line 164, authors write that “happiness in the 164 workplace is beneficial for both employees and organizations” – which has been empirically proven in the past. In the next lines (165-167), authors argue the benefits of the self-leadership, which should be the part of hypotheses development.

Authors are suggested to rewrite the “Introduction” so as to keep the relevant portions building the overall research context, elucidating the purpose that the research is trying to fulfil. Authors may choose to create a separate heading explaining the variables of the study in detail. For example, lines 58-63 highlight the beneficial effects of self-leadership, followed by the explanation of the mechanism in the next paragraph. The next paragraph (lines 84-94) takes the entire narrative back to the advantages of self-leadership, which takes the readers back to the lines 58-63.

2. Hypotheses under Investigation

I was expecting better and nuanced argumentation here supporting the hypotheses. Authors should highlight the individual hypotheses, building upon the previous literature and their own understanding of the substantive variables of the study. My suggestion is the bring some part of the ‘Introduction’ to this section which potentially may strengthen the overall arguments.

The argumentation for hypotheses 3-4 is based upon a few studies (e.g., Marques-Quinteiro et al., 2019) only. The authors finally ‘predict’ the hypotheses. To my mind, I do not find enough support for the hypotheses 3-4 in the manuscript, and based upon that, author argue in lines 199-201 about the hypotheses, which appears the pretty intuitive. Authors are strongly suggested to strengthen this part.

The overall conceptual model could have been presented to give a better understanding of the hypothesized relationships.

3. Materials and methods

The data was collected in 2016-2020. Though the retrospective analysis was approved by the ethics committee (I do not question that), the entire training was provided following Marques-Quinteiro et al. (2019) (line 233). This chronology is difficult to understand. Authors should explain that how they collected data from a program in 2016, when the training provided was based on a 2019 study.

It is a good practice to provide the in-line citation for methods used (e.g., line 360).

4. Results

Authors should explain the probable reasons behind the non-statistical significance of some of the results (e.g., self-leadership contrast between 3-4). Same goes for happiness also, where half the times, the results were not statistically significant (p-value > 0.05).

5. Discussion

Lines 527-560 are not appropriately placed in the discussion. Line 561 onwards, the findings of the study are discussed. Hypothesis 1 is an important finding of the study, however, I have concerns about the lines 564 – 566 because the study did not examine the ‘strategies’ precisely. The same is reflected in the authors' interpretation of the results also with the word ‘most probably’ (line 564).

Lines 580-593 are avoidable as it takes the readers back to the methodology (e.g., choice of LMM in line 581, line 590).

Authors should discuss the hypotheses individually (e.g., line 594). Hypotheses 2 and 3 are supported together, though in the hypotheses development phase, those were conceptualized differently. Regarding non-supported hypothesis 4, the explanation could be made stronger conceptually, and not just because of the process of data collection. Authors are suggested to look beyond the methods to explain this part.

Overall, this work is of sound relevance to handle the autonomy and self-leadership for the employees working in different organizations. I am hopeful that authors would find the suggestions helpful in developing this study better.
